# Identifying latent classes of physical activity profiles over time among adolescents in Ontario, Canada

**DOI:** 10.1186/s12889-024-18280-9

**Published:** 2024-03-19

**Authors:** M. Claire Buchan, Sarah A. Richmond, Kelly Skinner, Scott T. Leatherdale

**Affiliations:** 1 School of Public Health Sciences, University of Waterloo, Waterloo, ON N2L 3G5 Canada; 2 Dalla Lana School of Public Health, University of Toronto, Toronto, ON M5T 3M7 Canada; 3 Public Health Ontario, Toronto, ON M5G 1V2 Canada

**Keywords:** Physical activity, Physical education, Sport participation, Adolescent, Repeated measures latent class analysis

## Abstract

**Background:**

Physical activity behaviours are known to be highly correlated. Adolescents who participate in one type of physical activity (e.g., physical education) have a greater likelihood of participating in other physical activities (e.g., organized sports); however, little research has examined participation rates in various physical activity behaviours concurrently. This study identified longitudinal physical activity profiles among secondary school aged youth in Ontario, Canada.

**Methods:**

We used data from the COMPASS Study, a school-based prospective cohort study of adolescents in Canada. Using a repeated measures latent class analysis, Ontario students who participated in grade 9 PE in 2015-16 were analysed through to 2018-19 (*n* = 1,917). Latent classes were defined by: PE participation, guideline adherence (≥ 60 min/day of moderate to vigorous activity over the last 7 days), and sport participation (varsity, community, and/or intramural). Multinomial logistic regression models were used to examine associations between latent class membership and student characteristics.

**Results:**

Three distinct latent classes were identified for females and four were identified for males. These classes were: (1) Guidelines (high probability of guideline adherence; females: 44%; males: 16%), (2) PE & Sports (high probability of PE and sport participation; females: 33%; males: 43%), (3) Guidelines & Sports (high probability of guideline adherence and sport participation; females: 23%; males: 23%;), and (4) Inactive (low probability of all physical activity indicators; males: 18%). Strength training, sleep, and English grade were associated with class membership among females. Ethno-racial identity, weekly spending money, strength training, and English and math grades were associated with class membership among males.

**Conclusions:**

Findings suggest that latent physical activity profiles differ by sex. Guideline adherence was the most common class among females, indicating high levels of independent physical activity, whereas PE & Sport participation was the most common class among males, indicating greater tendency towards organized activities. Additionally, a substantial number of male students were not engaging in any physical activity. Participation in both PE and sports did not necessarily lead to meeting physical activity guidelines, highlighting that these activities alone may not be providing sufficient levels of physical activity that align with current recommendations for Canadian youth.

## Introduction

Adolescence is a critical developmental period during which many behavioural patterns are established [1]. Health behaviours, including physical activity patterns, are set during adolescence and track into adulthood [2]. The World Health Organization (WHO) and the Canadian Society for Exercise Physiology (CSEP) recommend that adolescents accumulate ≥ 60 min of moderate-to-vigorous physical activity (MVPA) daily and incorporate vigorous physical activities as well as muscle and bone-strengthening activities ≥ 3 days per week [3, 4]. However, evidence suggests that youth are not engaging in suggested levels of regular physical activity, and have grown increasingly sedentary in the last decade [5]. Schools have been identified as an ideal setting for addressing physical inactivity among adolescents, given that they spend majority of their waking hours at school [6]. Opportunities for physical activity that are embedded within the school curriculum, such as physical education (PE) courses, can provide equal opportunity for youth of all ages and backgrounds to structure activity into their weekly routine [1, 7, 8].

Many PE programs are developed with the intention of fostering physically literate adolescents who are active both inside and outside of school settings [1, 9, 10]. Despite this, most secondary schools in Ontario, Canada do not mandate annual PE courses after grade 9, precipitating a substantial drop in PE participation rates with increasing age [7]. A similar age-related decline is seen globally and is more pronounced among female students; among the estimated 20–65% of secondary school students globally who are not enrolled in regular PE classes past grade 9 [7, 8, 11–13], girls comprise a larger proportion compared to boys [12, 14, 15]. In Canada, PE is developed and implemented at the provincial level; provincial and territorial Ministries of Education are responsible for overseeing the policies, curriculum, and operation of public education across each province and territory [16]. As such, the PE policy in Ontario is unique from those of other provinces, territories, regions, and countries.

Physical activity behaviours are known to be highly correlated; existing studies have demonstrated that previous physical activity participation [17] and community sports participation [18–20] are positively associated with current physical activity patterns in adolescence. Additionally, adolescents who participate in one type of physical activity (e.g., PE) have a greater likelihood of participating in other physical activities (e.g., organized sports) [21]. Further, diverse participation in physical activity during adolescence is associated with higher levels of physical activity in adulthood [22–24]. Despite the need for a comprehensive approach to examine concurrent participation in various physical activity behaviours over time, PE participation research to date has primarily examined PE participation in isolation, without consideration of other co-occurring physical activity behaviours, and has been descriptive in nature [12, 21, 25, 26].

Data-driven statistical methods, such as latent class analysis and cluster analysis, are becoming increasingly common approaches to identifying connections between health behaviours [27–30]. Latent class analysis is a person-centred statistical approach that identifies clusters of participants that share common characteristics [29]. Repeated measures latent class analysis (RMLCA) is an extension of the traditional latent class analysis, permitting the examination of class indicators over multiple time points [31]. In the context of PE and physical activity research, RMLCA allows for the identification of common patterns of behaviour change (i.e., physical activity profiles), and the probability of engaging in those behaviours over time.

To better understand age-related decline in physical activity during adolescence, this research investigated longitudinal physical activity profiles (non-mandatory PE participation, adherence to physical activity guidelines, and sport participation) among a large sample of secondary students in Ontario, Canada. This study had the following objectives: (1) to identify the latent classes of physical activity profiles among secondary school aged adolescents over time; (2) to determine whether the identified latent classes differ between female and male adolescents; and (3) to determine which behavioural characteristics differentiate the latent classes.

## Methods

### Design

This study used data from the *Cohort study of obesity, marijuana use, physical activity, alcohol use, smoking and sedentary behaviour* (COMPASS Study), a school-based prospective cohort study (2012–2021) of adolescents in Canada. The COMPASS study collects student- and school-level data from schools in Alberta, British Columbia, Ontario, and Quebec, Canada using a purposive sampling approach and an active-information passive-consent parental permission protocol. All students attending participating schools were eligible to participate and could withdraw at any time. The COMPASS study received ethics clearance from the University of Waterloo Office of Research Ethics (ORE: #30,118) as well as participating school boards. A detailed description of COMPASS study methods is available online (www.compass.uwaterloo.ca) or in print [32].

### Participants

This study used linked-longitudinal data from students in Ontario who participated in four consecutive years of the COMPASS Study (Time 1 (T1): 2015–16, *n* = 37,106; Time 2 (T2): 2016-17, *n* = 34,078; Time 3 (T3): 2017-18, *n* = 31,654; Time 4 (T4): 2018-19, *n* = 30,675) [16]. Student data were linked across timepoints using a unique, self-generated code as described in COMPASS data collection protocols [33]. A total of 2,036 students from 48 secondary schools across Ontario were successfully linked from T1 to T4. Students who were not enrolled in grade 9 at T1 (*n* = 110) and those who did not report their sex at T1 (*n* = 9) were excluded.

### Measures

A detailed description of the development and selection of the measures used in the COMPASS questionnaire has been previously published and is available online (www.compass.uwaterloo.ca) [34].

#### Physical activity

Physical activity measures included self-reported PE participation, guideline adherence, and sports participation. The COMPASS questionnaire does not measure the quantity of physical activity performed during various physical activity contexts (intramural, community, or varsity sports, and PE), rather it captures participation in each activity within a given academic school year.

#### PE participation

was assessed by asking students to select one of the following response options: “Yes, I am taking one this term,” “Yes, I will be taking one or have one this school year, but not this term,” or “No, I am not taking a physical education class at school this year.” Responses were collapsed to a binary variable indicating PE participation or not during a given school year.

#### Physical activity guideline adherence

was assessed using a modified version of the physical activity measure in the School Health Action Planning and Evaluation System (SHAPES) [34]; the COMPASS MVPA measure has been found to have acceptable test-retest reliability and criterion validity [35]. Students were asked to report the number of hours (0–4) and minutes (0–45) of both moderate (e.g., walking, biking to school, and recreational swimming) and hard (e.g., jogging, team sports, and jump-rope) physical activity on each of the last 7 days. Responses were totaled, averaged, and dichotomized into two groups “<60 minutes on each of the last 7 days” and “≥ 60 minutes on each of the last 7 days” to align with the MVPA component of the WHO and CSEP guidelines [3, 4].

#### Sport participation

was assessed through three distinct questions. Students were asked to report their participation in each intramural, community, and varsity sports as “Yes,” “No,” or “None offered at my school.” A composite binary variable for overall sport participation was created; those reporting participation in at least one of the three sport programs were classified as sport participators.

#### Other physical activity measures

Students were asked to report the number of days they engaged in **strength training** exercises. Responses were categorized into 0 days, 1–3 days, or 4–7 days. **Sedentary behaviour** was assessed by asking students to report the number of hours (0–9) and 15-min increments (0-45 min) they spend engaging in various sedentary following activities (e.g., watching/streaming TV shows or movies, playing video/computer games, doing homework, talking on the phone, surfing the internet, and texting, messaging, or emailing,). Estimates across each activity, except “doing homework” were totaled, and averaged to provide a measure of hours of daily sedentary behaviour. **Active transportation** was assessed by asking students to report their method of travel both to and from school. Those who reported using active transportation (e.g., walk or bicycling) at least one way were classified as using active transportation.

#### Sleep

##### Sleep

was assessed by asking students to report the number of hours and minutes they typically sleep each night. Responses were totalled, averaged, and reported as a binary indicator of the CSEP sleep guideline adherence (≥ 8 h per night) [4].

#### Academic indicators

Students were asked to report their approximate overall mark in their current or most recent **English** and **Math courses**. Responses for each course were categorized into < 60%, 60–80%, 80–90%, and > 90%. **Desired educational attainment** was assessed by asking students “What is the highest level of education you would like to get?” Response options included: “Some high school or less”, “High school diploma or graduation equivalency”, “College/trade/vocational certificate”, “University Bachelor’s degree”, “Graduate school”, or “I don’t know”.

#### Sociodemographic measures

Students were asked to self-report their **sex** (female, male) and **age** (13–18 years). Students were asked to self-report their **ethno-racial identity**. Responses were recategorized as a dichotomous indicator of racialized (Black, Asian, Latin American or Hispanic, Mixed, Other) or non-racialized (White). As a proxy for student socioeconomic status, students were asked to report their **weekly spending money** ($0, $1–$20, $21–$100, $100+, Don’t know). This measure has been previously demonstrated to be a more accessible value for youth to report on than household income [36].

### Analysis

Descriptive statistics at T1 were examined for the entire sample and stratified by sex. Differences in baseline characteristics between females and males were compared using chi-square and t-tests, where appropriate. An RMLCA was used to identify latent classes characterized by different longitudinal patterns of physical activity indicators across four consecutive years of secondary school. A RMLCA was selected for this analysis rather than a latent transition analysis (LTA) as patterns of responses across all time points could be characterized in the latent classes (rather than examining probabilities of transitioning from a particular latent class at time 1 to another latent class at time 2) and it permitted a larger number of repeats (i.e., all 4 years of secondary school) [29]. Categorical indicators of PE participation, guideline adherence, and sport participation were used as the latent class indicators. Each binary indicator was measured at four time points (T1-T4, corresponding to grades 9, 10, 11, and 12). Sex was used as a grouping variable to explore the differences in the latent class structure between females and males. Models accounted for the nesting of students within schools by taking into account the non-independence of observations due to cluster sampling [37]. The number of latent classes were selected using various measures of model fit (relative: Akaike Information Criterion (AIC) and Bayesian Information Criterion (BIC); between-model: Lo–Mendell–Rubin Adjusted Likelihood Ratio Test). Alongside model fit indicators, parsimony and interpretability of the model were considered when selecting the number of latent classes.

Sex-stratified multinomial logistic regression models were used to examine student characteristics associated with class membership. A considerable amount of literature has established that numerous student characteristics and health behaviours influence physical activity, PE enrollment, and/or sports participation among adolescents [12, 15, 18, 38]. Student characteristics and health behaviour variables available in the COMPASS database that are known or suspected to influence at least one of the physical activity indicators in the latent class models were included in this analysis. Student characteristics included age [39, 40], ethno-racial identity [18, 20], weekly spending money [18], use of active transportation [41], sedentary behaviour [18, 42], sleep [43], strength training [44], desired educational attainment [45], English grade [46], and math grade [46]. RMLCA models were conducted using MPlus version 8 and logistic regression analyses using SAS version 9.4.

## Results

### Study participants

Overall, 1,917 students were included in this study. Approximately half of the sample was female (53%), and the majority (77%) were 14 years of age at T1 (Table 1). Weekly spending money, minutes of MVPA, guideline adherence, and varsity and community sport participation differed between males and females at T1. Male students in this sample engaged in more minutes of MVPA (115.7 vs. 96.4 min/day) compared to female students. A greater proportion of males met the physical activity guidelines (55% vs. 42%), and participated in varsity (47% vs. 41%) or community sports (62% vs. 50%) compared to females.

**Table 1:** Sample descriptives by sex at T1 (2015/16)

	Total sample (*n* = 1,917) n (%)	Females (*n* = 1,011) n (%)	Males (*n* = 906) n (%)	Chi-square /t	*P* value
**Age**					
13	90 (5)	45 (4)	45 (5)	0.317	0.957
14	1473 (77)	778 (77)	695 (77)		
15	348 (18)	185 (18)	163 (18)		
16	6 (0)	3 (0)	3 (0)		
**Ethno-racial identity**					
Non-racialized	1407 (73)	742 (73)	665 (73)	0.0167	0.897
Racialized	500 (26)	262 (26)	238 (26)		
Missing	10 (0)	7 (1)	3 (0)		
**Weekly spending money**					
Zero	476 (25)	254 (25)	222 (24)	11.200	**0.024**
$1-$20	786 (41)	425 (42)	361 (40)		
$21-$100	319 (17)	157 (15)	162 (18)		
$100+	63 (3)	22 (2)	41 (4)		
Don’t know	260 (13)	145 (14)	115 (13)		
Missing	13 (1)	8 (1)	5 (0)		
**PE participation**					
Yes	952 (50)	500 (49)	452 (50)	0.266	0.876
Yes, but not this semester	755 (39)	396 (40)	359 (40)		
No	191 (10)	104 (10)	87 (10)		
Missing	19 (1)	11 (1)	8 (1)		
**MVPA (daily)**					
Minutes (median, IQR)	107.1 (64.3–154.3)	96.4 (62.1–147.9)	115.7 (72.9–160.7)	4.106	**< 0.0001**
Missing	90 (5)	40 (4)	50 (5)		
**Guideline adherence (past week)**					
Yes	926 (48)	428 (42)	498 (55)	30.634	**< 0.0001**
No	953 (50)	562 (55)	391 (43)		
Missing	38 (2)	21 (2)	17 (2)		
**Strength training**					
0 days	361 (19)	189 (1)	172 (9)	2.680	0.444
1–3 days	826 (43)	452 (45)	374 (41)		
4–7 days	706 (37)	357 (35)	349 (38)		
Missing	24 (1)	13 (1)	11 (1)		
**Sedentary behaviour (daily)**					
Hours (median, IQR)	6.0 (4.0–8.5)	6.0 (3.75–8.5)	6.0 (4.0–8.5)	0.388	0.698
Missing	80 (4)	46 (4)	34 (4)		
**Varsity sports**					
Yes	841 (44)	416 (41)	425 (47)	6.807	**0.009**
No	1058 (55)	587 (58)	471 (52)		
Missing	18 (1)	8 (1)	10 (1)		
**Community sports**					
Yes	1073 (56)	506 (50)	567 (62)	30.969	**< 0.0001**
No	830 (43)	498 (49)	332 (37)		
Missing	14 (1)	7 (1)	7 (1)		
**Intramural sports**					
Yes	803 (42)	417 (41)	386 (43)	0.338	0.561
No	1098 (57)	585 (58)	513 (57)		
Missing	16 (1)	9 (1)	7 (1)		
**Sport participation**					
Yes	1370 (71)	683 (67)	687 (76)	16.05	**0.0003**
No	539 (28)	323 (32)	216 (24)		
Missing	8 (0)	5 (0)	3 (0)		

Note: IQR = interquartile range. MVPA = moderate-to-vigorous physical activity. PE = physical education. Bolded values indicate significance

### Model selection

Model fit statistics for RMLCAs with one to five classes were considered (Table 2). A three-class model was selected as the best fitting model among females, whereas a four-class model was selected as the best fitting model among males. Models with the lowest model selection criteria, a statistically significant Lo-Mendell-Rubin test, and the most appropriate interpretability were selected for this study.

**Table 2:** Model fit indices for 1–5 latent class models among students who participated in COMPASS

Number of Classes	Log-Likelihood	FP	AIC	BIC	LMRT	Entropy
					*p*-Value	
**Female** (***n*** **= 1011)**						
1	-7450.1	12	14924.1	14983.1	-	1.00
2	-6701.1	25	13452.1	13575.1	0.00	0.87
**3**	**-6550.7**	**38**	**13177.3**	**13364.2**	**0.03**	**0.79**
4	-6467.7	51	13037.4	13288.3	0.34	0.74
**Male** (***n*** **= 906)**						
1	-6678.595	12	13381.2	13438.9	-	1.00
2	-5931.161	25	11912.3	12032.5	0.00	0.86
3	-5764.971	38	11605.9	11788.7	0.04	0.80
**4**	**-5676.066**	**51**	**11454.1**	**11699.4**	**0.02**	**0.79**
5	-5607.787	64	11343.6	11651.4	0.24	0.77

Note: AIC = Aikake Information Criterion. BIC = Bayesian Information Criterion. FP = free parameters. LMRT = Lo–Mendell–Rubin Test. Bolded models were selected

### Class description

**Fig. 1:**
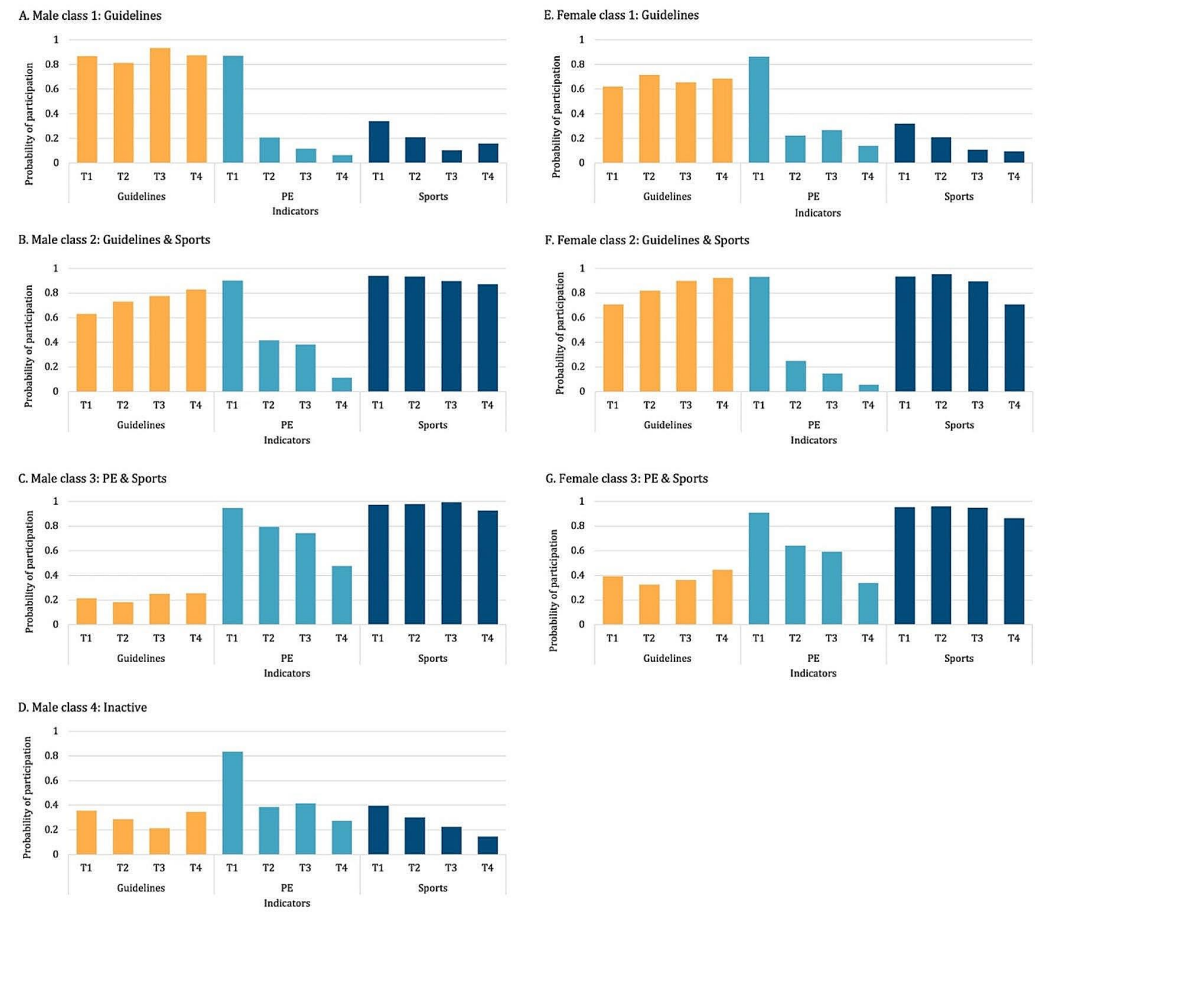
Physical activity profile item probabilities for each latent class among students who participated in COMPASS

The three classes identified among female adolescents in this study, characterized by their clustered physical activity profiles, were labeled: *Guidelines*, *Guidelines & Sports*, and *PE & Sports*. All three classes identified among females were also identified among male students. One additional class, labeled *Inactive*, was identified among males. Item response probabilities for each physical activity indicator in each class among female and male adolescents are presented graphically in Fig. 1.

The first latent class, “*Guidelines*”, represented 44% of female adolescents and 16% of male adolescents. This class was characterized by consistent adherence to the physical activity guidelines and no participation in sports or PE across timepoints. The “*PE & Sports*” class, which represented 33% and 43% of females and males, respectively, was characterized by consistent participation in sports across timepoints, participation in PE in grades 10 and 11, but no adherence to the physical activity guidelines at any timepoint. The “*Guidelines & Sports*” class is characterized by consistent adherence to the physical activity guidelines and sport participation across all timepoints, but no participation in PE in grades 10–12. This class represented 23% of both female and male adolescents. Finally, the “*Inactive*” class, which represented 18% of male adolescents, was characterized by no adherence to the physical activity guidelines or participation in sports across all timepoints, and no participation in PE in grades 10–12.

### Regression analyses

Results of the logistic regression models for female and male adolescents are presented in Tables 3 and 4, respectively.

Among female adolescents, strength training of any frequency was positively associated with being in the *Guidelines & Sports* class, compared to the *Guidelines* class. Conversely, daily duration of sedentary behaviour was negatively associated with being in the *Guidelines & Sports* class. Strength training of any frequency, engaging in active travel, having weekly spending money between $21–100, and meeting the sleep guidelines were positively associated with being in the *PE & Sports* class, compared to the *Guidelines* class. Conversely, being racialized and having an English grade > 90% were negatively associated with being in the *PE & Sports* class.

Among male adolescents, strength training 4–7 days a week and having weekly spending money between $21–100 were positively associated with being in the *Guidelines & Sports* class, compared to the *Guidelines* class. Conversely, being racialized and daily duration of sedentary behaviour were negatively associated with being in the *Guidelines & Sports* class. Similarly, strength training of any frequency, having weekly spending money between $21–100, and meeting the sleep guidelines were positively associated with being in the *PE & Sports* class, compared to the *Guidelines* class. Being racialized, daily duration of sedentary behaviour, having a desired educational attainment of secondary school or less, and having an English grade > 60%, however, were negatively associated with being in the *PE & Sports* class. Finally, strength training of any frequency, having weekly spending money between $21–100, and having an English grade between 60 and 80% were positively associated with being in the *Inactive* class, and being racialized and having a desired educational attainment of graduate school were negatively associated with being in the *Inactive* class.

**Table 3:** Student characteristics associated with latent class membership among female students who participated in COMPASS

	Model 1: Class 2 (Guidelines & Sports) vs. Class 1 (Guidelines)	Model 2: Class 3 (PE & Sports) vs. Class 1 (Guidelines)
	OR (95% CI)	OR (95% CI)
**Age**		
	0.78 (0.53–1.15)	0.95 (0.67–1.34)
**Ethno-racial identity**		
Racialized	0.89 (0.59–1.35)	**0.59 (0.40–0.87)**
Non-racialized (ref)	-	-
**Weekly Spending Money**		
$0	0.93 (0.59–1.46)	0.94 (0.62–1.43)
$1–20 (ref)	-	-
$21–100	1.13 (0.65–1.95)	**1.73 (1.08–2.78)**
$100+	1.99 (0.61–6.47)	1.34 (0.41–4.35)
Don’t know/missing	0.99 (0.57–1.71)	1.18 (0.72–1.92)
**Strength Training**		
0 (ref)	-	-
1–3 days	**3.02 (1.73–5.26)**	**2.28 (1.35–3.83)**
4–7 days	**3.01 (1.78–5.09)**	**4.06 (2.53–6.52)**
**Active Travel**		
Yes	0.85 (0.54–1.35)	**0.49 (0.31–0.77)**
No (ref)	-	-
**Sedentary Behaviour (hours, per day)**		
	**0.89 (0.85–0.95)**	0.96 (0.92–1.01)
**Sleep Guidelines**		
Yes	1.15 (0.80–1.65)	**1.53 (1.10–2.13)**
No (ref)	-	-
**Desired Educational Attainment**		
Secondary School or less	0.26 (0.03–2.26)	0.76 (0.22–2.60)
College or Trade	0.82 (0.32–2.07)	0.87 (0.40–1.88)
Bachelors (ref)	-	-
Graduate School	1.61 (0.91–2.85)	1.56 (0.93–2.61)
I don’t know	0.80 (0.42–1.51)	0.71 (0.40–1.26)
**English Grade**		
< 60%	0.65 (0.06–6.52)	1.28 (0.27–6.12)
60–80%	0.70 (0.43–1.13)	1.04 (0.69–1.56)
80–90% (ref)	-	-
> 90%	0.66 (0.42–1.04)	**0.61 (0.39–0.94)**
**Math Grade**		
< 60%	0.33 (0.11–1.01)	0.49 (0.22–1.12)
60–80%	0.83 (0.52–1.34)	1.07 (0.71–1.62)
80–90% (ref)	-	-
> 90%	1.46 (0.94–2.26)	1.31 (0.86–2.00)

Note: CAD = Canadian dollars. CI = confidence interval. OR = odds ratio. PE = physical education. Ref = reference category. Bolded values indicate significance

**Table 4:** Student characteristics associated with latent class membership among male students who participated in COMPASS

	Model 3: Class 2 (Guidelines & Sports) vs. Class 1 (Guidelines)	Model 4: Class 3 (PE & Sports) vs. Class 1 (Guidelines)	Model 5: Class 4 (Inactive) vs. Class 1 (Guidelines)
	OR (95% CI)	OR (95% CI)	OR (95% CI)
**Age**			
	0.95 (0.58–1.56)	0.90 (0.55–1.45)	1.22 (0.70–2.11)
**Ethno-racial identity**			
Racialized	**0.50 (0.30–0.85)**	**0.25 (0.15–0.42)**	0.72 (0.41–1.27)
Non-racialized (ref)	-	-	-
**Weekly Spending Money**			
$0	1.03 (0.58–1.82)	0.72 (0.41–1.26)	1.06 (0.56–2.01)
$1–20 (ref)	-	-	-
$21–100	**2.27 (1.05–4.91)**	**2.32 (1.11–4.84)**	**2.46 (1.08–5.58)**
$100+	2.49 (0.70–8.80)	1.38 (0.40–4.77)	1.31 (0.31–5.51)
Don’t know/missing	**2.82 (1.09–7.28)**	**2.86 (1.15–7.11)**	**2.87 (1.05–7.82)**
**Strength Training**			
0 (ref)	-	-	-
1–3 days	1.02 (0.54–1.93)	**2.98 (1.52–5.86)**	**2.10 (1.03–4.28)**
4–7 days	**1.80 (1.00–3.21)**	**11.03 (5.91–20.57)**	**3.22 (1.65–6.28)**
**Active Travel**			
Yes	1.28 (0.70–2.35)	1.06 (0.58–1.92)	1.32 (0.68–2.56)
No (ref)	-	-	-
**Sedentary Behaviour (hours, per day)**			
	**0.93 (0.86–1.00)**	**0.93 (0.87–0.99)**	0.98 (0.91–1.06)
**Sleep Guidelines**			
Yes	1.44 (0.88–2.35)	**1.64 (1.02–2.62)**	1.32 (0.77–2.27)
No (ref)	-	-	-
**Desired Educational Attainment**			
Secondary School or less	0.24 (0.05–1.10)	**0.27 (0.07–0.98)**	0.75 (0.22–2.55)
College or Trade	0.7 (0.25–1.93)	0.91 (0.36–2.30)	1.07 (0.40–2.84)
Bachelors (ref)	-	-	-
Graduate School	0.77 (0.36–1.66)	0.55 (0.26–1.17)	**0.33 (0.14–0.78)**
I don’t know	0.85 (0.38–1.92)	0.76 (0.35–1.65)	0.66 (0.28–1.56)
**English Grade**			
< 60%	0.84 (0.19–3.70)	0.81 (0.22–3.01)	1.26 (0.30–5.37)
60–80%	1.35 (0.75–2.45)	1.17 (0.67–2.04)	**2.28 (1.20–4.34)**
80–90% (ref)	-	-	-
> 90%	0.67 (0.34–1.31)	**0.34 (0.17–0.68)**	0.57 (0.23–1.39)
**Math Grade**			
< 60%	0.44 (0.13–1.48)	0.69 (0.24–1.96)	0.65 (0.20–2.10)
60–80%	0.57 (0.30–1.07)	0.84 (0.47–1.51)	0.92 (0.48–1.76)
80–90% (ref)	-	-	-
> 90%	1.42 (0.75–2.67)	1.09 (0.58–2.06)	1.13 (0.52–2.42)

Note: CAD = Canadian dollars. CI = confidence interval. OR = odds ratio. PE = physical education. Ref = reference category. Bolded values indicate significance

## Discussion

This study examined latent physical activity profiles over time among a large sample of Ontario secondary school aged adolescents. Using a RMLCA, three and four unique physical activity profiles were identified among female and male adolescents, respectively. Three common patterns emerged among both female and male adolescents: *Guidelines*, *PE & Sports*, and *Guidelines & Sports*. A fourth latent class was identified only among male adolescents: *Inactive*. To the best of our knowledge, this is the first study to examine longitudinal patterns of PE participation within the context of other co-occurring physical activity indicators.

### Latent classes

Our findings suggest that longitudinal physical activity profiles differ by sex. The most prevalent class among females was *Guidelines*, representing nearly half of female adolescents in this study. Physical activity participation in the *Guidelines* class was characterized by consistent guideline adherence without PE or sports participation, indicating high levels of independent physical activity. By contrast, the most prevalent class among male adolescents was *PE & Sports*, indicating a greater tendency towards organized activities. This observed difference suggests a sex-based preference for certain physical activity types. These findings align with previous sex- and gender-based physical activity literature demonstrating that a larger proportion of female adolescents tend to participate in individual physical activities (e.g., running, walking, fitness classes), whereas a larger proportion of male adolescents tend to participate in team-based sports [47, 48]. Additionally, previous literature has consistently identified higher PE participation rates among male adolescents compared to their female counterparts [12, 14]. Our findings extend previous research by suggesting that sex-based differences in physical activity participation exist across a variety of physical activity indicators examined concurrently.

Interestingly, a large proportion of adolescents in this study (33% of females, 43% of males) demonstrated consistent participation in both PE and sports but were not meeting physical activity guidelines, suggesting that these activities alone do not constitute sufficient levels of physical activity to align with current recommendations for adolescents (i.e., ≥ 60 min of MVPA/day [3]). Either the frequency of participation (days/week) or the duration of MVPA (min/day) while participating in these activities was not sufficient to meet recommendations. These findings are consistent with a recent systematic review demonstrating that youth only spend about 40% of a typical PE lesson engaging in MVPA [6]. Similarly, a recent study reported that while sport participants were more likely to meet physical activity guidelines compared to their non-participating counterparts, a large proportion were not meeting the guidelines [49]. Notably, adolescents in the *Guidelines & Sports* class consistently met physical activity guidelines while participating in organized sports, indicating it is possible for adolescents to meet recommendations by participating in sports, either alone or in combination with independent physical activity, if the frequency or duration is sufficient. These findings may reflect variation in the types of sport participation. Adolescents participating in community or varsity sports, with compulsory games, practices, and workouts throughout the week, may be more likely to meet guidelines than those enrolled in irregular or intramural sport programs.

Unique to male adolescents, an *Inactive* class was identified. Roughly one in five male adolescents in this study were not engaging in any type of physical activity. This contradicts research on sex-based patterns of physical activity behaviours suggesting lower levels of physical activity among female adolescents across all behaviours compared to males [17, 39, 50]. Male adolescents in this class should be considered the highest risk group with respect to physical inactivity, as they did not participate in any type of physical activity behaviour beyond their compulsory grade 9 PE class. Future research should explore reasons for non-participation and strategies to overcome barriers to participation among this group of male adolescents.

### Characteristics of classes

Another objective of this study was to determine which student-level characteristics were predictive of class membership. We observed a strong relationship between strength training and class membership among both female and male adolescents. Adolescents who engaged in strength training were more likely to be in the *PE & Sports* and the *Guidelines & Sports* classes. The strongest association was observed between participating in 4–7 days per week of strength training and being in the *PE & Sports* class. These findings are consistent with previous research indicating that strength training activities are particularly common among adolescents involved in sport participation [51, 52], as strength training activities are often key components of weekly training programs for many competitive sports teams. Interestingly, strength training of any frequency among males was associated with being in the *Inactive* class. While somewhat unexpected, these findings are consistent with previous research indicating that strength training has become increasingly popular among adolescents not involved in sports, possibly as a means to increase muscularity [51]. Further research into the intentions behind strength training and how participation impacts other physical activity indicators is warranted. We also observed an association between hours of sedentary behaviour and class membership among both female and male adolescents. Students who engage in more sedentary behaviours were less likely to belong to the *Guidelines & Sports* (among both females and males) or *PE & Sports* (males only) classes. These results align with previous research demonstrating that greater physical activity participation is associated with less sedentary behaviour [42].

Racialized youth were less likely to belong to the PE & Sports and Guidelines & Sports classes, consistent with previous literature demonstrating that PE and sports participation often differs by race or ethnicity [53, 54]. These findings must be considered within the context of intersectionality. Race, gender, socio-economic status, ability, and other socio-demographic factors play an important role in shaping an individual’s access, experience, and attitudes towards physical education, physical activity, and sport [55]. Equitable opportunity for all youth requires that physical activity programming be receptive to diverse social identities and attentive to ways in which youth are differentially impacted by policies and practices [55].

### Strengths and limitations

We applied sex-stratified RMLCA models to a large cohort of adolescents in Ontario, Canada who participated in four consecutive years of the COMPASS study. RMLCA analyses permitted the examination of class indicators over multiple time points, allowing for identification of common patterns of behaviour change.

This study has a few limitations that should be noted. Firstly, student-level COMPASS data are not nationally representative, and as such, our findings may not be generalizable to all adolescents in Ontario. COMPASS was not designed to collect nationally representative data, instead relying on purposive sampling procedure to ensure robust sample sizes. Active-information passive-consent data collection procedures yield high participation rates and reduced selection bias [56]. We advise readers to consider the influence of differing populations, curricula, and implementation strategies between school boards, regions, and countries when generalizing the findings from this study. Secondly, data for this study were collected using self-report questionnaires. Self-report data may be influenced by recall bias or social desirability bias and could result in an overestimation of true physical activity levels [57]. It is possible that this overestimation could have influenced latent class identification and class membership estimates. The questionnaires used in the COMPASS study, however, have been previously validated as reliable measures of physical activity in youth [35]. Thirdly, the self-report ethno-racial identity question used in this study was selected for inclusion in the COMPASS student questionnaire in 2012 and captures the constructs of both race and ethnicity [58]. For this study, this variable has been operationalized as “ethno-racial identity” to align with the recently published guidance on the reporting of race and ethnicity by the Canadian Medical Association Journal [58]. To create more inclusive and specific options for participants, the question was updated for the 2020–2021 COMPASS data collection cycle. Fourth, the PE and sport participation questions used in this study do not take into consideration the frequency of participation and the quantity of physical activity performed during these physical activity contexts. This could result in a heterogeneity among students reporting participation in PE and sports and could have influenced the findings of this study. Finally, COMPASS does not collect data on physical literacy, attitudes towards physical activity types, or intention for participation in physical activity. Previous research suggests that physical activity enjoyment and intentions are strong indicators of physical activity engagement [59–61] necessitating future research to explore their impact on class membership.

### Implications

The findings from this study have important implications for adolescent health research and public health policy. Alarmingly, a large proportion of male adolescents in this study were not consistently engaging in any type of physical activity beyond their mandatory grade 9 PE requirements. PE provides a unique opportunity to reach a large population of children and adolescents, particularly those who may not be able to participate outside of school [62]. The pre-existing infrastructure of PE positions it to be one of the most cost-effective population-level health-promotion strategies targeting adolescents [63]. The implementation of compulsory PE programming throughout secondary school may be a key first-step in promoting physical and health literacy and ultimately physical activity among youth; however, findings from this study suggest that adolescents who consistently participate in PE do not necessarily meet physical activity recommendations. The impact of physical activity participation extends beyond meeting the current recommendations; physical activity participation, when delivered in appropriate frequency, intensity, and context, can positively influence mental health outcomes [64–66]. It remains unknown, however, whether the quantity of physical activity accrued during PE is associated with favourable mental health outcomes. Future research should aim to evaluate the impact of PE participation on physical and health literacy as well as physical activity levels, mental health, and overall wellbeing.

Our findings also indicated that physical activity profiles over time varied by sex; male adolescents had a greater tendency to engage in organized sport and female adolescents had a greater tendency to engage in independent physical activities. While many PE curricula in Canada are beginning to deviate from sport- and game-focused learning, the time lag between curriculum design and development to implementation and culture change [67] could partially explain the low PE participation rates, particularly among female adolescents. Our findings may provide some justification for expanding the variety of activities offered in PE programs. However, a better understanding of how the variety of activities provided during PE lessons influences participation rates is required.

## Conclusion

This study investigated longitudinal physical activity profiles in a large sample of adolescents in Canada. Our findings reaffirm that physical activity profiles among adolescents are clustered and appear to vary by sex. In light of declining rates of physical activity among youth today, these findings can help inform public health programming, particularly within the school context. The bodies that govern curriculum decision-making in Canada, such as the Ministries of Education, should consider implementation of mandatory PE programming for all students throughout secondary school, with special care taken to ensure types of activities offered suitable and accessible for all youth. Future research quantifying the impact of PE and sports participation on overall physical activity levels and wellbeing is warranted.

## Data Availability

The datasets generated and analyzed during the current study will not currently be shared because this is an ongoing study; however, access to the data supporting the findings of the study can be requested at https://uwaterloo.ca/compass-system/information-researchers.
